# Buffalo Hospital of the Sisters of Charity

**Published:** 1848-12

**Authors:** 


*Buffalo Hospital of the Sisters of Charity.*—We are happy to state that this charitable Institution is in successful operation. About sixty patients have already been received. Additions to the building are in progress, and farther enlargements in contemplation.

Medical students are admitted to visit the wards with the attending Surgeon and Physician, Wednesday and Saturday of each week, on payment of a small fee for the benefit of the hospital. Clinical lectures by the attending Physician and Surgeon will be given on Saturdays, which are to be exclusively devoted to clinical instruction, no other lectures being given at the College on that day.

